# Cultural and personal channels between religion, religiosity, and corruption

**DOI:** 10.1016/j.heliyon.2023.e16882

**Published:** 2023-06-05

**Authors:** Yaron Zelekha, Gil Avnimelech

**Affiliations:** Faculty of Business Administration, Ono Academic College, Kiryat Ono, Israel

**Keywords:** Corruption, Culture, Institutions, Religion, Religiosity, Trust

## Abstract

Literature on the effect of religion and religiosity on corruption has failed to distinguish between direct and mediated effect and to determine the different roles of two alternative mediation channels—the personal versus the cultural channel. Using a data set of 102 countries, we found that hierarchical religions have significant positive associations with corruption levels, while the non-hierarchical Protestant Christianity has significant negative associations with corruption levels. Most of the effect is mediated by the cultural channel. However, some hierarchical religions (particularly Sunni Islam and Orthodox Christianity) have both an indirect mediated effect and a direct unmediated effect, suggesting an additional dissociative parameter besides the hierarchical/non-hierarchical parameter, which we suggest is the status of a formal/informal state religion. The findings are closely linked to the modern view of economic development that although institutions give rise to economic growth, it is culture that shapes institutions.

## 1 Introduction

Although significant efforts have been made to combat corruption, it remains a prevalent issue worldwide, with a negative impact on economic development and growth [[1], [2], [3], [4], [5]]. According to Transparency International corruption is the abuse of entrusted power for private gain,^1^ while the World Bank defines corruption as the abuse of public office for private gain.^2^ Research on the causes of corruption highlights the crucial role of institutions in creating a conducive environment for corruption to thrive. The factors shaped by institutions that contribute to corruption include democracy, particularly the effective separation of powers [[6], [7], [8]], the freedom of the press [[8], [9], [10]], transparency, accountability [8,11], the rule of law and effective judicial systems [[12], [13], [14], [15]], and the level of competition and the ease of doing business [14,[16], [17], [18]], which reduce corruption. On the other hand, high levels of government intervention in the economy and regulation increase corruption [16,[19], [20], [21]]. Cultural factors also play a significant role in corruption, with societies characterized by high of power distance and collectivism being associated with higher levels of corruption [[22], [23], [24], [25], [26], [27], [28]].

Religion plays a significant role in influencing corruption levels [26,[29], [30], [31], [32], [33]]. While all religions encourage good moral conduct and ethical behavior, studies show that different religions are associated with varying levels of corruption. Notably, countries whose primary religions are hierarchical religions such as Catholic Christianity (Catholicism), Eastern Orthodox Christianity, and Islam, tend to have higher corruption levels, particularly in comparison to Protestant Christian countries [21,30,[34], [35], [36], [37]]. Supporting this claim, [30] found that corruption levels are lowest in countries with a Protestant majority and highest in countries with an Orthodox Christian majority.

Recent research suggests that religious devotion, or religiosity, is associated with higher levels of corruption, in addition to the effect of each religion [29,38]. While [32] found that higher levels of religious freedom reduce corruption, and several scholars, including [29,37,39] argue that religious diversity and freedom, which promote competition among religions, also reduce corruption.

The existing literature on the effects of religion on corruption faces several issues. First, most studies fail to distinguish between the dominant religion and the percentage of each religion in a given population. Therefore, it remains unclear whether membership in minority religions is associated with the same level of corruption as in countries where these religions are dominant. This issue could help identify the channel that confers the associations between religion and corruption. If, indeed, members of minority religions are associated with the level of corruption characterized by the dominant religion (and not with the level of corruption in countries in which their religion is dominant), it may be a country's general religious norms that affect its culture and that these norms are distributed not only to the dominant religion's members (the cultural channel). On the other hand, if members of minority religions are associated with the levels of corruption within countries in which their religions are dominant; this may be the effect of the religious norms that are distributed only to the religion's members (the personal channel). In fact, there are indications that the dominant religion may culturally affect members of minority religions, such as the effect of different religions on entrepreneurship [[40]^41^,^42^], which supports the cultural channel view over the personal channel view.

Second, the literature has not resolved whether the cultural effect of religions on corruption is transmitted through institutions, norms, or both. Although hierarchical religions were associated with high corruption levels, it is unclear whether the effect is transmitted only through the role of institutions (for example, democracy, government economic intervention, and competition), or the result of normative transmitters (that is, the norms of members of the specific religion).

Third, recent research found that religiosity is associated with higher corruption levels, possibly due to personal channels [29]. However, this outcome may also have resulted from omitted normative and institutional variables.

To address these issues, we analyzed a dataset of 102 countries, covering 89% of the world's population, and posit that the cultural channel supersedes the personal channel, and suggest that members of minority religions adopt the ethical conduct of the dominant religion, even after controlling for a large set of institutional and normative variables. Furthermore, we argue that hierarchical religions contribute to high levels of corruption both directly and through their effect on institutions and norms. Similarly, we argue that formal or informal state religions also contribute directly to high levels of corruption. Finally, we find that religiosity has an additional contribution to corruption levels through its interconnectivity with hierarchical religions, especially hierarchical state religions. Overall, our findings support the cultural over the personal channel view, which is closely linked to the modern view of economic development which posits that although institutions give rise to economic growth, it is culture that shapes institutions.

This paper makes several significant theoretical contributions to the existing literature. First, it examines the role of minority religions, allowing for a comparison between the cultural effect of the country's dominant religion and that of minority religion. Second, it accesses the different roles of the cultural and personal channels in the relationship between religion, religiosity, and corruption. Third, it is the first study, to the best of our knowledge, to isolate the roles of religiosity, hierarchical religions, and state religions, which can resolve the internal contradiction between advocating for better moral conduct associated with religiosity and the association of religiosity with corruption. It also addresses the difference between the association of the non-state hierarchical Catholic religion and corruption versus that of state hierarchical religions and corruption. Thus, this study offers a revised theoretical model of religion, religiosity, and corruption.

The rest of this paper is structured as follows. In Section 2, we present a comprehensive review of the literature, discussing how religion can create a cultural environment for corruption, while religiosity can play a role with ambiguous outcomes. In this section, we develop our hypotheses. Section 3 describes our unique data set and method. In Section 4, presents the empirical results. In Section 5, we discuss our conclusions and policy implications.

## 2 Literature review and general hypotheses

Religions are often associated with advocating for better moral conduct and improved ethical behavior at a personal level [29,33]. Furthermore, religion serves as a constant reminder of the distinction between good and evil and the importance of moral conduct [32,42]. If the personal channel were the only channel through which religions influenced society, we would likely find a negative relationship between religiosity and corruption.

However, the relationship between religiosity and corruption is more complex. For example, [29] found that religiosity level is positively associated with corruption level. This finding is attributed to the idea that as individuals become more devout, they may demand less accountability and transparency from their religious leaders and institutions, which can lead to corruption. This is also related to findings in the literature on culture and corruption, which found that more collectivist societies are often more corrupt [22,28]. Moreover, religious authorities may not condemn political corruption if it brings them additional benefits or power [29].

To develop a more comprehensive understanding of the relationship between religion and corruption, it is essential to distinguish between the personal and cultural channels. While an inverse relationship between religiosity and corruption might be expected, if the personal channel were the only way religion affected society, previous studies have demonstrated otherwise [23]. To determine whether the cultural channel, rather than the personal channel, is the primary channel to corruption, this study will examine both the dominant religion in a country (representing the cultural channel) and the percentage of each religion in the population (representing the personal channel). Previous research has typically investigated only one of these variables, which limits comprehension of the relationship between religion and corruption and might explain why the personal channel has not been distinguished from the cultural channel. We argue that the cultural channel has a greater impact on the association between religion and corruption than the personal channel. Consequently, we hypothesize that the dominant religion in a country has a stronger and more significant association with the level of corruption than the percentage of a particular religion in the population. Our hypothesis is as follows.

Hypothesis (1) H1: The impact of the dominant majority religion dummy variable on the level of corruption scores will be stronger and more significant than the impact of the variable of the percentage of a specific religion in the population when estimating the association of specific religions with the level of corruption.

### 2.1 Hierarchical religions and corruption

Recent research has indicated that countries with hierarchical religions tend to have higher levels of corruption than countries with non-hierarchical religions [21,30,36]. This may be due to the institutionalized relationship between hierarchical religious institutions and the state, which creates a conflict of interest when it comes to monitoring and criticizing public corruption [21,38]. In many European countries, for example, the Catholic Church and the Eastern Orthodox Church receive government funds or are involved in political parties, while in many Muslim countries, religious leaders are appointed by the state and receive state financing [29]. This intertwined relationship can foster greater tolerance of corruption within hierarchical religious institutions [33].

Furthermore, hierarchical religions tend to be less “democratic” than non-hierarchical religions, in the sense that there are no “competing” churches, and religious leaders hold more power [42]. This concentration of power can lead to greater corruption, as Lord Acton wrote, “All power corrupts, and absolute power corrupts absolutely”. Moreover, in countries where the majority religion is hierarchical and where the religiosity level of its adherents is high, religious leaders and institutions may hold even greater power and influence, which increases the risk of corruption. This is also consistent with findings from the literature on culture and corruption, which suggest that societies characterized by high power distance tend to have higher levels of corruption [22,27,28]. Thus, we hypothesize the following.

Hypothesis (2a) *H2a*: When hierarchical religions are characterized as dominant majority religions, they are positively associated with corruption.

Hypothesis (2b) *H2b*: In hierarchical religions, the level of religiosity in a country is positively associated with corruption.

### 2.2 State religions and corruption

However, the parameter of hierarchical religions is not the only factor that distinguishes world religions. Protestant Christianity, for example, is not only non-hierarchical; it is anti-hierarchical, as it is characterized by individualism and tends to monitor public officials [21,43]. Consequently, the corruption level tends to be lower in countries with higher rates of Protestants [21,44]. While Orthodox Christianity, Sunni Islam, and Shia Islam differ from Hinduism and Buddhism on the parameter of hierarchical religions, they also share their positions as formal or informal state religions. Catholicism and Protestant Christianity, on the other hand, are rarely, if ever, considered the formal or sole religion of their countries [21]. Moreover, Protestantism often leads to an independent civil society and strict separation of church and state [21]. In contrast, Orthodox Christianity, Hinduism, Buddhism, and Sunni and Shia Islam religions are often considered the formal and sole religion of the state, while other religious practices are accepted but not an element of the state itself [21].

Islam was established as a ruling religion, in which its founder is the religious leader as well as the leader of the empire. Although the political leadership in Muslim countries was separated from the religious leadership throughout history, Islam still considered itself to be directing the state, and both the people and the political leaders cooperated with religious leaders to receive public support and acknowledgment. This unique relationship has led scholars to coin the term “political Islam” [45,46]. Orthodox Christianity also has a long tradition of involvement with state affairs since its origins in the Byzantine Empire. As a leading scholar indicated: “The Church was not a department of state. But it was closely integrated into the daily life of an empire which was regarded as being ideally the mimesis or copy of the heavenly kingdom” [47].

In fact, both Islam [45,46] and Orthodox Christianity [47] have developed their hierarchical characteristics as a result of the historical intermingling of state and religion. Even in the former Soviet Union, where religion was suppressed during the Communist era, the church became again highly involved with the state again after 1991, as seen in countries like Russia [48] and Romania [49]. In contrast, Catholicism developed its hierarchical structure as an international organization led by the Pope, headquartered in an independent Vatican state, rather than an independent and separate element belonging to each state.

Therefore, there may be a more complex relationship between hierarchical religions that are also a formal state religion (or at least considered an important element of the state) and corruption scores, in which rather than just an indirect correlation through the cultural channel, it will also have a direct link to corruption. We expect that this formal position allows hierarchical religions to have more active involvement in the public sector, but it may also weaken their willingness to monitor and criticize public corruption due to the intertwined institutions of local religions and states. Additionally, as mentioned earlier, a decrease in religious freedom and diversity is associated with an increase in corruption [29,32,37]. Countries with state religions often have lower levels of religious freedom and diversity. Thus, we hypothesize the following.

Hypothesis (3a) H3a: The state religions, when characterized as dominant majority religions, are negatively associated with the level of corruption scores (positively associated with corruption).

Hypothesis (3b) H3b: In state religions, the religiosity level in a country is negatively associated with the level of corruption scores (positively associated with corruption).

Hypothesis (3c) H3c: In hierarchical state religions (Sunni Islam, Shia Islam, and Orthodox Christianity), the religiosity level's negative association with level of corruption scores is higher than in non-hierarchical state religions or in hierarchical non-state religions.

### 2.3 Normative and institutional aspects of corruption

Religions not only transmit cultural effects through institutional differences but also through significant normative and sociocultural differences that can affect corruption. Research indicates that more religious individuals have a high level of trust in others [50], in the government, and are less willing to break the law [51]. However, [52] found that the level of religiosity reduces trust in others and this negative association increases with the degree of religious diversity. Moreover, hierarchical religious institutions have been associated with decreased levels of trust [52], which can lead to an emphasis on vertical relationships at the expense of more egalitarian and cooperative ties, ultimately resulting in higher levels of corruption [36,53].

Attitudes toward “sins” and forgiveness also play a role. Protestant Christianity and Judaism believe that avoiding sins is the responsibility of individuals during their lives, while Catholicism may be viewed as more tolerant toward sins and the possibility of redemption [54]. Additionally, there is a long tradition in the literature that suggests that Jewish and Protestant Christian norms regarding the punishment of sins in one's current life contributed to the famous “Protestant work ethic” that played a critical role in the formation of entrepreneurial activities, the spirit of capitalism, and people's economic behaviors [55]. Furthermore, Catholic and Muslim countries have been found to perform lower in education [56,57], which can be associated with a lesser ability to monitor public institutions and, thus, be related to more corruption [15].

To determine whether both normative and institutional differences contribute to different levels of corruption, we use a large set of normative and institutional controls and examine whether they significantly mediate the dominant religions’ contribution to corruption. Thus, we hypothesize the following.

Hypothesis (4a) H4a: The associations between hierarchical state religions (Sunni Islam, Shia Islam, and Orthodox Christianity), when characterized as dominant majority religions, and the level of corruption scores will be negatively significant even after controlling for norms and institutional transmitters.

Hypothesis (4b) H4b: The negative associations between hierarchical and/or state religions, when characterized as dominant majority religions, and the level of corruption scores are significantly mediated by both the normative and institutional transmitters.

**Fig. 1:**
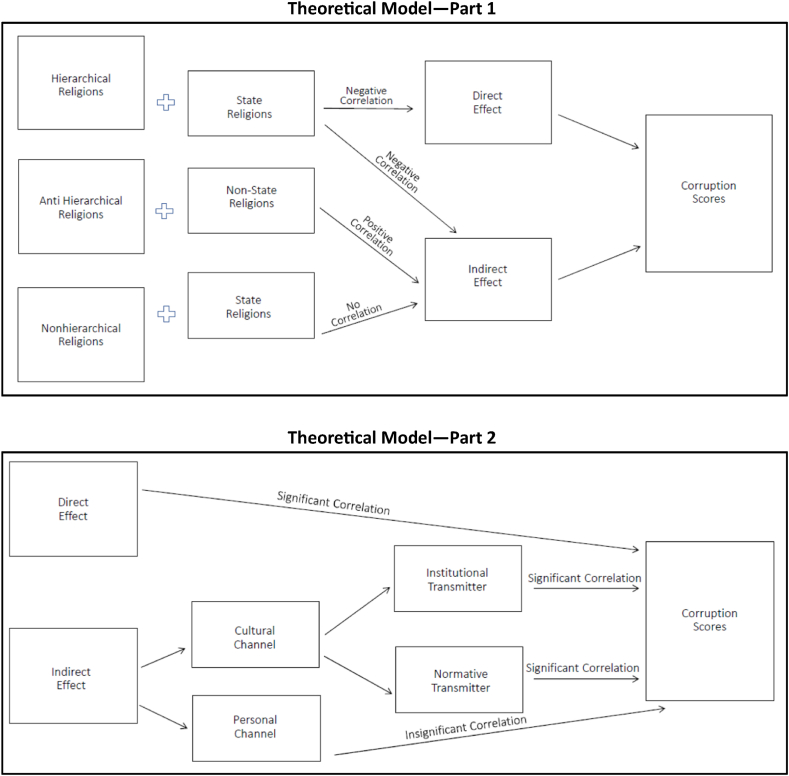
Theoretical model.

Our theoretical model is illustrated in Fig. 1, while Table 1 presents the typologies of religions based on hierarchical and state dimensions.

**Table 1:** Types of religions.

Religion	Hierarchical	State
Sunni Muslim	Hierarchical	Yes
Shia Muslim	Hierarchical	Yes
Christians Catholic	Hierarchical	No
Christians Orthodox	Hierarchical	Yes
Christians Protestant	Anti-Hierarchical	No
Hindu	Non-hierarchical	Yes
Buddhist	Non-hierarchical	Yes

## 3 Dataset and methods

The study sample comprises 102 countries, which account for 89% of the world population in 2020 (these countries represent a total population of 6.86 billion people).

### 3.1 Dependent variable

Corruption is estimated using the Transparency International Corruption Perception Indexes (TI CPI) for the year 2020. The TI CPI ranks 180 countries based on how corrupt their public sectors are perceived to be. The index combines surveys and assessments of corruption collected by various reputable institutions, making it the most widely used corruption indicator worldwide. Measuring absolute levels of corruption in countries is challenging, given that corruption is primarily a hidden illegal activity. Thus, assessing the relative levels of corruption across countries by capturing perceptions about corruption is the most reliable method. The TI CPI provides an expert-based perception of corruption levels that may influence investors and consumer behavior.

To assess the robustness of the results, we also used the World Bank's Worldwide Governance Indicators (WGI) corruption control rank as a complementary measure. The WGI corruption control rank measures the perceived level of corruption based on data collected from a variety of sources. The WGI is produced by the World Bank and provides a range of governance indicators for over 200 countries. The corruption control rank measures the extent to which public power is exercised for private gain, including both petty and grand forms of corruption. The rank is based on a composite score that takes into account multiple data sources, including surveys of experts, businesspeople, and citizens, as well as assessments by international organizations and other publicly available information. The corruption control rank ranges from 0 to 100, with higher scores indicating a lower perceived level of corruption. It is important to note that the WGI corruption control rank is a perception-based measure, and therefore may not reflect actual levels of corruption in a country. Additionally, the data used to calculate the rank may be subject to biases and limitations. Despite these limitations, the WGI corruption control rank remains a widely used tool for comparing corruption levels across countries and tracking changes over time.

Despite similarities between the two measures, the CPI score and the WGI corruption control rank differ in some respects. The CPI relies solely on expert assessments and opinion surveys of businesspeople and analysts from around the world, while the WGI incorporates a wider range of data sources, including citizen surveys and assessments by international organizations. Additionally, while the CPI focuses specifically on corruption in the public sector, the WGI corruption control rank measures the perceived level of corruption more broadly, including both petty and grand forms of corruption.

### 3.2 Independent variables

The purpose of this study is to investigate the effect of religion on corruption utilizing three sets of variables. First, a dummy variable is used to identify the primary religion of each country, including the eight major religions (Protestant Christianity, Catholicism, Orthodox Christianity, Sunni Islam, Shia Islam, Judaism, Buddhism, and Hinduism). Additionally, a dummy variable is added to represent countries where the primary religion is not one of these major religions, primarily African Christian countries and Pagan countries. Second, the study examines the percentage of the population belonging to each of the eight major religions, as well as the percentage of individuals who do not belonging to any religion. Data for the first two sets of variables were obtained from the CIA Factbook.

The third set of variables concerns the level of religiosity, and data were obtained from three sources. First, the World Values Survey (WVS) provided data for the seventh wave for 79 countries as of December 31, 2021, and 14 additional countries for the sixth wave. Second, the Gallup International Association published data at the end of 2016 for nine countries (out of 66) not included in the WVS. The level of religiosity was estimated using ten questions from the WVS survey, which measured the importance of religion in people's lives. These questions were used to create a total religion grade, adjusting the scales to measure the more religious side. They included questions such as V9 (“For each of the following, indicate how important it is in your life: Religion”); V25 (“Now I am going to read off a list of voluntary organizations. For each organization, could you tell me whether you are an active member, an inactive member or not a member of that type of organization? Church or religious organization”); V145 (“Apart from weddings and funerals, about how often do you attend religious services these days?“); and V152 (“How important is God in your life?“). The survey used different Likert scales and “yes-or-no” questions. Combining the 10 questions allowed for a single total religion importance grade. A factor analysis for the 10 questions was conducted, revealing only one significant factor associated with all of the items. Results indicated a high reliability of the estimated total religion importance grade, as indicated by the very high Cronbach's α of the entire questionnaire (0.945). The total religion grade of the 14 countries obtained from WVS6 and the nine countries obtained from Gallup was estimated using a similar procedure of incorporating questions related to religion into a single grade. Fixed effects dummy variables for each data set were included in each specification.

### 3.3 Control variables

The study includes control variables based on previous research on the macroeconomic drivers of corruption. These variables consist of economic freedom, democracy index, government expenditure on education, urbanization, population growth rate, GDP (PPP) per capita, gender discrimination parameters, the level of trust in other people, and dummy variables indicating countries with a colonial or socialist history, which are associated with corruption. The level of economic freedom and the democracy index were included because centralized decision making and the concentration of power are potential causes of corruption, while greater market competition reduces corruption [14,16,58]. Government expenditure on education was included because education can help address corruption in the long run as more educated electorates can better monitor government activities [15,59]. Urbanization and population growth rates were included because it has been suggested that increased population concentration in urban areas increases competition for favors and willingness to break the law [60,61]. GDP (PPP) per capita was included because economic development raises rent-seeking costs and thereby reduces corruption [62].

Furthermore, we included several normative and cultural variables, such as gender discrimination parameters (gender ratios in labor force participation, gender parity of school enrollment, and the proportion of seats held by women in national parliaments) and the level of trust in other people, as culture has also been discussed in the literature as a possible source of corruption [22,63]. Some previous studies found a significant positive relationship between masculinity and corruption [26,27,64]. Ethnic diversity was also included as societies with greater diversity may experience more conflict and competition for resources, which can lead to corruption [65,66]. Additionally, dummy variables indicating countries with colonial and socialist history were added as they were found to be associated with corruption [8,21]. All data descriptions, sources, and statistics are given in Table 1. The categories of different religions according to hierarchical and state dimensions appear in Table 2a.

**Table 2A:** Summary statistics.

Variable	Average/Share	SD	Median	Data Sources
**Dependent Variables (main and for robustness tests)**				
CPI Scores	48.65	19.31	44	Transparency International^a^
WGI Corruption Score	53.40	30.04	52.64	World Bank's WGI^b^
**Independent Variables**				
% of Sunni Muslims	0.20	0.32	0.02	CIA Factbook^c^
Sunni Muslims Majority	22/102	–	–	
% of Shia Muslims	0.03	0.13	0.00	
Shia Muslims Majority	3/102	–	–	
% of C. Catholics	0.30	0.36	0.06	
C. Catholics Majority	36/102	–	–	
% of Christians Protestants	0.13	0.22	0.01	
C. Protestants Majority	8.5/102	–	–	
% of Christians Orthodox	0.11	0.25	0.00	
C. Orthodox Majority	11/102	–	–	
% of Hindus	0.01	0.08	0.00	
Hindu Majority	1/102	–	–	
% of Buddhists	0.05	0.18	0.00	
Buddhist Majority	6/102	–	–	
% of Jews	0.01	0.07	0.00	
Jewish Majority	1/102	–	–	
% of Non-Religious	0.14	0.18	0.05	
Religiosity	44.06	17.97	43.67	WVS and Gallup Int. Association^d^
**Control Variables**				
General Trust in People	24.47	16.99	21.19	World Values Survey (WVS)^e^
Colonial & Socialist History	74/102	–	–	CIA Factbook
Democracy Score	6.01	2.21	6.44	Economist Intelligence Unit^f^
Economic Freedom Score	7.16	0.93	7.36	Fraser Institute^g^
GDP per Capita US$	27,156	21,586	20,383	World Bank World Development Indicators^h^
% of Urban Population	67.09	19.31	68.50	
% of Women in Parliament	26.55	11.82	26.19	
Gini Index 2018	35.76	12.32	18.60	
Population Growth (%)	0.77	0.91	0.72	
Gov. Expenditure on Edu.	0.70	0.20	0.76	
Ease of Doing Business	68.0	51.3	54.5	

* If the year of the data is not explicitly stated, it should be noted that the data pertains to the year 2020.

a Transparency International. (2020). Corruption Perceptions Index 2020. Retrieved from https://www.transparency.org/en/cpi/2020/index/nzl.

b World Bank's Worldwide Governance Indicators 2020 (WGI 2020). Retrieved from https://databank.worldbank.org/source/worldwide-governance-indicators#.

c Central Intelligence Agency (2022). In The World Factbook. Retrieved from https://www.cia.gov/the-world-factbook/countries/.

d Gallup International Association (GIA). Retrieve from https://gallup-international.com/survey-results-and-news/survey-result/more-prone-to-believe-in-god-than-identify-as-religious-more-likely-to-believe-in-heaven-than-in-hell.

e World Values Survey (WVS) seventh wave (2021). Retrieved from https://www.worldvaluessurvey.org/WVSDocumentationWV7.jsp.

f Economist Intelligence Unit (2021) The Democracy Index. Retrieve from https://www.eiu.com/n/campaigns/democracy-index-2021/.

g Fraser Institute (2022). The Economic Freedom Index. Retrieve from https://www.fraserinstitute.org/studies/economic-freedom.

h World Bank – DataBank. Retrieve from https://databank.worldbank.org/source/world-development-indicators.

**Table 2B:** Correlation between religion and corruption.

Variable	CPI Scores	WGI Corruption Score
% of Sunni Muslims	−0.39	−0.41***
Sunni Muslims Majority	−0.38	−0.39***
% of Shia Muslims	−0.27***	−0.30***
Shia Muslims Majority	−0.20**	−0.23**
% of C. Catholics	0.05	0.08
C. Catholics Majority	0.03	0.06
% of C. Protestants	0.48***	0.41***
C. Protestants Majority	0.41***	0.36***
% of C. Orthodox	−0.08	−0.06
C. Orthodox Majority	−0.09	−0.08
% of Hindus	−0.06	−0.03
Hindu Majority	−0.04	−0.02
% of Buddhists	0.05	0.06
Buddhist Majority	0.07	0.07
% of Jews	0.06	0.06
% of non-Religious	0.37***	0.38***
Religiosity	−0.65***	−0.63***

***p < .01; **p < .05, *p < .1.

### 3.4 Dataset limitations

Although our data set allowed us to examine 89% of the world population, it only incorporated 102 out of 195 recognized countries. Notably, most of the excluded countries are small in size, which introduces bias towards larger countries and limits our ability to investigate differences between very small and larger countries. Nonetheless, we controlled for both GDP and population in our specifications to account for potential confounding factors.

Furthermore, our data sources did not enable us to examine changes over time. Although the scope of the WVS Survey has increased over time, we still needed to use two waves of the WVS survey and the Gallup International Association data set to build our sample. Using previous waves to build data for longitudinal research would have significantly limited our sample. We faced similar challenges with the corruption data sources. However, we believe that the inability to consider changes over short to medium terms is not crucial. This is because corruption, and by no means religion, tends to be highly stable according to the literature [67]. In fact, countries tend to settle into one of two stable equilibria over time: either with a high level of income and low corruption or with low income and high corruption [67]. This phenomenon is an expected outcome of the critical mass characteristics of the network economy, which characterizes corruption [68]. Therefore, longitudinal research will need to consider very long-time scales, which the present data sources do not yet allow.

## 4 Results

To test our hypotheses, we conducted four sets of regressions. The first set of regressions (Table 3a) examines the impact of each specific religion on corruption and estimates which type of religious variable is more significant in explaining corruption in a country: religion majority dummy or the percentage of a population a specific religion (Models A1, A2, A4 and A4 versus A5, A6, A7, and A8). We use two sets of models to perform this assessment. Models A1, A2, A4, and A4 include all eight religions as independent variables. Conversely, models A5, A6, A7, and A8 exclude religion variables that were not statistically significant in at least one model. The second set of regressions (Table 3b) aims to examine the impact of hierarchical and state religions in explaining corruption, both as dummy variables and as the dummy variable multiplied by the religiosity level. We also separated the effect of Protestant and Catholic majorities in some specifications. The third set of regressions (Table 3c) investigates the impact of the majority religion for each specific religion, while controlling for institutional and normative variables. The fourth set of regressions, as presented in Table 3d, investigates the impact of hierarchical and state religions, while controlling for institutional and normative variables.

**Table 3A:** OLS Estimation of Equations Explaining the Effect of Religion on Corruption Scores. Religion percentage in population vs. majority (w/o religiosity) - uncontrolled specifications.

DV: Corruption Score	A1	A2	A3	A4	A5	A6	A7	A8	A9 (A3′)	A10 (A7′)
Religiosity	–	−0.60*** (−6.23)	−0.58*** (−6.29)	–	–	−0.54*** (−5.99)	−0.55*** (−6.20)	–	−0.84*** (−5.70)	−0.80*** (−5.76)
Sunni Majority (in A8 * Religiosity)	–	–	–	–	−25.16*** (−4.94)	−10.66** (−2.14)	−9.68** (−2.15)	−0.46*** (−6.70)	–	−17.09** (−2.40)
% Sunni (in A4 * Religiosity)	−23.66*** (−2.93)	−0.54 (−0.07)	−2.35 (−0.37)	−0.49*** (−5.45)	–	–	–	–	−9.19 (−0.90)	–
Shia Majority (in A8 * Religiosity)	–	–	–	–	−32.10*** (−3.21)	−23.27*** (−2.69)	−22.29*** (−2.67)	−0.67*** (−3.68)	–	−41.46*** (−3.14)
% Shia (in A4 * Religiosity)	−36.17** (−2.52)	−19.20 (−1.55)	−22.14* (−1.93)	−0.77*** (−3.36)	–	–	–	–	−43.33* (−2.36)	–
Catholic Majority (in A8 * Religiosity)	–	–	–	–	−9.32** (−1.96)	−4.74 (−1.15)	−3.80 (−1.05)	−0.35*** (−4.23)	–	−5.78 (−1.01)
% Catholic (in A4 * Religiosity)	−10.81 (−1.33)	1.00 (0.14)	−1.90 (−0.36)	−0.51*** (−4.58)	–	–	–	–	−4.21 (−0.49)	–
Protestant Majority (in A8 * Religiosity)	–	–	–	–	19.66*** (2.97)	17.21*** (3.04)	18.02*** (3.35)	0.36** (2.37)	–	21.71** (2.55)
% Protestant (in A4 * Religiosity)	24.60** (2.40)	28.37*** (3.28)	25.53*** (3.54)	0.21 (0.90)	–	–	–	–	27.50*** (2.37)	–
Orthodox Majority (in A8 * Religiosity)	–	–	–	–	−15.22** (−2.45)	−11.35** (−2.12)	−10.41** (−2.11)	−0.40*** (−3.10)	–	−15.65** (−2.00)
% Orthodox (in A4 * Religiosity)	−11.14 (−1.23)	−3.00 (−0.39)	−5.71 (−0.89)	−0.43*** (−2.78)	–	–	–	–	−9.29 (−0.90)	–
Hindu Majority	–	–	–	–	−18.76 (0.55)	−3.27 (−0.23)	–	–	–	–
% Hindu	−18.58 (−0.90)	9.94 (0.56)	–	–	–	–	–	–	–	–
Buddhist Majority	–	–	–	–	−4.60 (−0.60)	−2.98 (−0.46)	–	–	–	–
% Buddhist	−4.30 (−0.37)	2.52 (0.26)	–	–	–	–	–	–	–	–
% Jews	10.51 (0.46)	22.03 (1.15)	–	–	–	–	–	–	–	–
Constant	56.28*** (8.83)	71.51*** (12.12)	73.23*** (15.25)	63.21*** (19.02)	58.76*** (14.86)	77.18*** (16.91)	76.42*** (18.09)	61.57*** (22.47)	92.45*** (11.98)	95.62*** (14.29)
N	102	102	102	102	102	102	102	102	102	102
$R^{2}$	0.36	0.55	0.54	0.37	0.39	0.56	0.56	0.40	0.50	0.53
${\bar{R}}^{2}$	0.30	0.50	0.51	0.34	0.34	0.52	0.53	0.36	0.46	0.50
S.E.	16.34	13.78	13.67	15.95	15.89	13.57	13.44	15.60	21.98	21.29

Notes: ***p < .01; **p < .05, *p < .1. (The values in brackets are t statistics).

Robustness tests: in models A9 and A10 the DV is WGI corruption control rank of the world bank rather than the CPI score from Transparency International.

**Table 3B:** OLS Estimation of Equations Explaining the Effect of Religion on Corruption Scores. Hierarchical and state religions - uncontrolled specifications.

DV: Corruption Score	B1	B2	B3	B4	B5	B6	B7	B8 (B1′)	B9 (b7′)
Religiosity	−0.54*** (−6.26)	−0.53*** (−6.38)	−0.55*** (−6.40)		−0.44*** (−4.30)	−0.48*** (−4.47)		−0.79*** (−5.97)	
Religion_freedom	16.53** (2.18)	11.88 (1.57)	17.30** (2.27)	16.94** (2.09)	16.94** (2.22)	20.23** (2.61)	17.41** (2.09)	28.45** (2.42)	30.26** (2.38)
Religiosity_Hierarchical_State_religion							−0.52*** (−6.75)		−0.81*** (−6.85)
Religiosity_Nonhierarchical_State_religion							−0.35*** (−3.19)		−0.49*** (−2.93)
Religiosity_ Hierarchical_Non-State religion							−0.48*** (−5.72)		−0.74*** (−5.75)
Religiosity_Nonhierarchical_Non-State_religion							0.01 (0.06)		−0.05 (−0.27)
Religiosity_Hierarchical Religions				−0.46*** (−6.37)	−0.18*** (−2.62)				
Religiosity_Hierarchical Religions besides Catholic						−0.20** (−2.50)			
Religiosity_Catholic						−0.20*** (−2.34)			
Religiosity_Non-Hierarchical Religions				−0.22** (−2.33)					
Dummy for Hierarchical_religion	−9.27*** (−2.87)	−6.29* (−1.88)						−13.24*** (−2.64)	
Dummy for Hierarchical Religions besides Catholic			−13.22*** (−3.42)						
Dummy for Catholic_majority			−8.03** (−2.29)						
Dummy for Protestant_majority		14.44** (2.63)							
State_religion Dummy	−5.92* (−1.95)	−5.57* (−1.88)		−9.03*** (−2.86)	−6.27** (−2.06)			−9.86** (−2.09)	
Constant	72.71*** (12.38)	71.43*** (12.48)	71.36*** (12.38)	61.24*** (10.54)	68.22*** (11.83)	66.08*** (11.29)	17.41*** (10.12)	87.18*** (9.55)	66.32*** (7.55)
N	102	102	102	102	102	102	102	102	102
$R^{2}$	0.52	0.56	0.52	0.46	0.52	0.49	0.45	0.51	0.45
${\bar{R}}^{2}$	0.50	0.53	0.50	0.43	0.50	0.47	0.42	0.49	0.42
S.E.	13.78	13.37	13.88	14.73	13.87	14.19	14.94	21.42	22.86

Notes: ***p < .01; **p < .05, *p < .1. (The values in brackets are t statistics).

Robustness tests: in models B8 and B9 the DV is WGI corruption control rank of the world bank rather than the CPI score from Transparency International.

**Table 3C:** OLS Estimation of Equations Explaining the Effect of Religion on Corruption Scores. Religion majority––controlled specifications.

DV: Corruption Score	C1	C2	C3	C4	C5	C6	C7	C8 (C2′)	C9 (C3′)
Sunni Majority (in C4 * Religiosity)		−7.83*** (−2.83)	−5.77** (−2.00)	−0.12** (−2.57)	−5.02* (−1.93)	−6.42** (−2.38)	−4.77* (−1.78)	−12.11*** (−2.83)	−10.82** (−2.36)
Shia Majority (in C4 * Religiosity)		−9.78* (−1.75)	−8.37 (−1.51)	−0.17 (−1.59)	−6.17 (−1.24)	−7.90 (−1.57)	−7.45 (−1.37)	−22.61** (−2.60)	−21.73** (−2.48)
Catholic Majority (in C4 * Religiosity)		−2.83 (−1.17)	−2.29 (−0.96)	−0.10* (−1.90)	−2.68 (−1.25)	−2.88 (−1.36)		−3.78 (−1.00)	−3.44 (−0.91)
Protestant Majority (in C4 * Religiosity)		−1.34 (−0.37)	−0.43 (−0.12)	−0.04 (−0.52)	−0.09 (−0.03)	−0.49 (−0.15)		−7.86 (−1.38)	−7.29 (−1.27)
Orthodox Majority (in C4 * Religiosity)		−8.06** (−2.46)	−6.77** (−2.06)	−0.17** (−2.36)	−4.91 (−1.67)	−5.43* (−1.84)	−5.56* (−1.86)	−14.35** (−2.82)	−13.54** (−2.61)
Religiosity			−0.14** (−2.06)		−0.14** (−2.17)	−0.09 (−1.45)	−0.15** (−2.18)		−0.09 (−0.82)
GDP_per_Capita					.0003*** (4.73)	.0003*** (4.47)			
Gini_Index						−0.24* (−1.70)			
Economic Freedom	7.41*** (5.72)	7.16*** (5.64)	6.52*** (5.08)	7.11*** (5.55)	4.31*** (3.48)	4.32*** (3.52)	6.44*** (5.07)	10.58*** (5.38)	10.18*** (5.01)
Democracy	3.26*** (5.51)	2.55*** (4.06)	2.68*** (4.33)	2.83*** (4.62)	2.53*** (4.55)	2.44*** (4.42)	2.69*** (4.39)	4.01*** (4.12)	4.09*** (4.17)
Women in Parliament	0.21** (2.49)	0.17** (1.98)	0.15* (1.81)	0.17* (1.96)	0.15** (2.01)	0.16** (2.11)	0.14* (1.73)	0.24* (1.80)	0.23* (1.70)
Population Growth	2.78** (2.50)	2.30* (1.89)	3.17** (2.50)	2.77** (2.30)	3.03*** (2.67)	3.36*** (2.95)	3.30** (2.63)	0.86 (0.45)	1.40 (0.70)
% Urban Pop.	0.27*** (5.00)	0.28*** (5.32)	0.25*** (4.70)	0.27*** (5.05)	0.12** (2.10)	0.15** (2.54)	0.26*** (4.83)	0.39*** (4.74)	0.37*** (4.36)
Trust in People	0.06*** (2.77)	0.06*** (2.66)	0.05** (2.13)	0.05** (2.27)	0.02 (1.04)	0.02 (0.86)	0.05** (2.35)	0.10*** (2.68)	0.09** (2.40)
Former Spanish Colony	−11.79*** (−3.90)	−12.42*** (−3.84)	−11.88*** (−3.72)	−10.89*** (−3.28)	−7.83** (−2.63)	−7.03** (−2.35)	−12.99*** (−4.40)	−19.17*** (−3.82)	−18.83*** (−3.73)
Gallup Dummy	−2.21 (−0.74)	−3.72 (−1.25)	−4.63 (−1.57)	−3.88 (−1.31)	−5.81** (−2.19)	−5.88** (−2.24)	−4.90 (−1.68)	−5.75 (−1.25)	−6.33 (−1.36)
Constant	−64.05*** (−7.07)	−53.23*** (−5.42)	−39.38*** (−3.35)	−51.64*** (−5.26)	−15.22 (−1.30)	−8.41 (−1.70)	−40.26*** (−3.60)	−94.80*** (−6.23)	−86.11*** (−4.63)
N	102	102	102	102	102	102	102	102	102
$R^{2}$	0.83	0.85	0.86	0.85	0.89	0.89	0.86	0.85	0.85
${\bar{R}}^{2}$	0.82	0.83	0.83	0.83	0.87	0.87	0.84	0.83	0.82
S.E.	8.37	8.10	7.96	8.11	7.13	7.05	7.91	12.57	12.59

Notes: ***p < .01; **p < .05, *p < .1. (The values in brackets are t statistics). Robustness tests: in models C8 and C9 the DV is WGI corruption control rank of the world bank rather than the CPI score from Transparency International.

**Table 3D:** OLS Estimation of Equations Explaining the Effect of Religion on Corruption Scores. Hierarchical and state religions––controlled specifications.

DV: Corruption Score	D1	D2	D3	D4	D5	D6	D7	D8 (D1′)	D9 (D7′)
Religiosity	−0.15** (−2.20)	−0.14** (−2.11)	−0.14** (−2.09)					−0.11 (−1.02)	
Religiosity_Hierarchical State							−0.12** (−2.12)		−0.16* (−1.85)
Religiosity_Hierarchical Non-State							−0.008 (−0.12)		0.06 (0.51)
Religiosity_Non-Hierarchical_State							−0.003 (−0.04)		−0.11 (−1.20)
Religiosity_Non-Hierarchical_Non-State							−0.08 (−1.33)		−0.03 (−0.26)
Religiosity_Hierarchical Religions				−0.09* (−1.89)	−0.09** (−2.25)				
Religiosity_Hierarchical Religions besides Catholic						−0.13*** (−3.16)			
Religiosity_Catholic						−0.10* (−1.82)			
Religiosity_Non-Hierarchical Religions				−0.01 (−0.17)					
Dummy for Hierarchical Religions	−3.61* (−1.79)	−3.79* (−1.81)						−5.26 (−1.61)	
Dummy for Hierarchical Religions besides Catholic			−6.20** (−2.09)						
Dummy for Catholic_majority			−2.20 (−0.98)						
Dummy for Protestant_majority		−1.17 (−0.33)							
State_religion Dummy	−2.27 (−1.04)	−2.29 (−1.04)		−3.55 (−1.63)	−3.53 (−1.63)			−5.93* (−1.67)	
Economic Freedom	6.58*** (5.22)	6.61*** (5.20)	6.58*** (5.27)	6.63*** (5.00)	6.69*** (5.26)	7.11*** (5.70)	6.78*** (5.05)	10.30*** (5.04)	10.27*** (4.84)
Democracy	2.85*** (4.80)	2.86*** (4.79)	2.71*** (4.52)	2.80*** (5.00)	2.78*** (4.66)	2.82*** (4.79)	2.93*** (4.77)	4.43*** (4.61)	4.70*** (4.84)
Women in Parliament	0.15* (1.81)	0.15* (1.82)	0.15* (1.91)	0.14 (1.60)	0.14 (1.63)	0.16* (1.96)	0.16* (1.83)	0.21 (1.58)	0.26* (1.89)
Population Growth	3.35*** (2.83)	3.35*** (2.82)	3.36*** (2.91)	2.62** (2.35)	2.58** (2.38)	3.14*** (2.93)	2.96*** (2.62)	1.87 (0.97)	2.03 (1.14)
% Urban Pop.	0.24*** (4.63)	0.24*** (4.61)	0.25*** (4.80)	0.27*** (5.08)	0.27*** (5.21)	0.27*** (5.18)	0.27*** (4.96)	0.33*** (3.87)	0.36*** (4.17)
Trust in People	0.05** (2.13)	0.05** (2.11)	0.05** (2.28)	0.06** (2.54)	0.06** (2.57)	0.05*** (2.41)	0.06** (2.57)	0.07* (1.91)	0.08** (2.24)
Former Spanish Colony	−11.06*** (−3.60)	−11.06*** (−3.58)	−11.98*** (−3.82)	−11.48*** (−3.64)	−11.44*** (−3.66)	−10.96*** (−3.34)	−10.94*** (−3.23)	−17.57*** (−3.53)	−15.95*** (−2.98)
Gallup Dummy	−4.21 (−1.45)	−4.27 (−1.46)	−4.61 (−1.59)	−3.86 (−1.31)	−3.82 (−1.30)	−3.77 (−1.29)	−3.44 (−1.15)	−5.17 (−1.10)	−4.57 (−0.96)
Constant	−38.47*** (−3.44)	−39.49*** (−3.39)	−40.01*** (−3.64)	−47.06*** (−4.22)	−47.82*** (−4.69)	−52.10*** (−5.51)	−52.25*** (−4.72)	−79.47*** (−4.38)	−95.07*** (−5.43)
N	102	102	102	102	102	102	102	102	102
$R^{2}$	0.85	0.85	0.86	0.85	0.85	0.85	0.84	0.84	0.83
${\bar{R}}^{2}$	0.84	0.84	0.84	0.83	0.83	0.83	0.82	0.82	0.81
S.E.	7.90	7.94	7.84	8.10	8.06	8.02	8.24	12.80	13.04

Notes: ***p < .01; **p < .05, *p < .1. (The values in brackets are t statistics).

Robustness tests: in models D8 and D9 the DV is WGI corruption control rank of the world bank rather than the CPI score from Transparency International.

To support hypothesis H1, we compared specifications A1, A2, A3, and A4 in Tables 3a and in which the independent variables were the percentages of each religion in a population, to specifications A5, A6, A7, and A8, in which the independent variables were dummies for each specific religious majority. Our findings revealed that using the religion majority dummy yielded higher R-squares in all specifications and that all t-values for each specific religion variable were more significant. This finding supports our hypothesis that the cultural channel of religious impact is more significant than the personal channel.

We tested associations between specific religions and levels of corruption using the eight major religions (A1 and A5), while controlling for the country's religiosity level (A2 and A6). We removed statistically insignificant religion variables in further specifications (A3 and A7), and the final specifications, we used specific religious variables multiplied by religiosity level (A4 and A8). Our results were consistent across all specifications and supported hypothesis H1.

The variables capturing the percentage share of Catholic Christians, Orthodox Christians, Hindus, Buddhists, and Jews in the population were found to be statistically insignificant both before and after controlling for religiosity level (Table 3a, Models A1 and A2). For Sunni Muslims, this variable was statistically insignificant only when controlling for religiosity level. The percentage share of Protestant Christians was positively significant with corruption score in the uncontrolled specification (Table 3a, Models A1, A2, and A3), but it lost its significance in the controlled specifications. The percentage share of Sunni Muslims was negatively significant with corruption score only in two uncontrolled specifications (Models A1 and A4).

In contrast, the dummy variables for most majority religions (Sunni Muslims, Shia Muslims, Orthodox Christians, and, in two specifications, also Catholic Christians) were negatively significant in the uncontrolled specifications (Models A5, A6, A7, and A8), and the Protestant Christians majority dummy was positively associated with the corruption score (negatively with corruption). The majority dummy variables for Hindus and Buddhists were found to be statistically insignificant, and the Jews majority variable was excluded from the model as it was equal to 1 in only a single case.

In the specifications that controlled for institutional and normative variables (Table 3c), only the majority dummies for Christian Orthodox and Sunni Muslim remained negatively significant across all specifications. Catholics (a non-state religion) and Shia Muslims remained negatively significant in only one specification.

In support of hypothesis H2a, we found that in specifications B1, B2, and B3, the dummy variables for hierarchical religions, when characterized as dominant majority religions, were always significantly associated with lower levels of corruption scores (higher levels of corruption) in uncontrolled specifications, regardless of their share in the population, including or excluding Catholics in this dummy, state religion, or Protestant majority. However, the share of hierarchical religions was not associated with decreased levels of corruption when they were not the dominant majority religions (as shown in specifications not presented in the paper).

In support of hypothesis H2b, we observed from specifications B4, B5, and B6 that the variable representing the religiosity level of hierarchical religions, when characterized as dominant majority religions, was significantly associated with lower levels of corruption scores (higher levels of corruption) in all uncontrolled specifications (with or without Catholics, and with or without a dummy for state religion), regardless of their share in the population or inclusion of the general country's religiosity level in the model (specifications B5 and B6).

In support of hypothesis H3a and hypothesis H3b, we included control variables for countries where a state religion is a majority (Models B1, B2, B4, and B5) and for countries where a hierarchical state religion is a majority (Model B3). We also added variables representing the religiosity level in countries where a state religion is a majority (Model B7) and where a hierarchical religion is a majority (Models B4, B5, and B6). In each of these uncontrolled specifications (Table 3b), all variables representing countries where a state religion is a majority were significantly negatively associated with corruption scores (indicating high levels of corruption), supporting hypotheses H2a and H3a. Similar results were found for the variable representing the religiosity level in countries where a state and hierarchical religion is a majority, supporting hypotheses H2b and H3b. Model B7 showed that the negative association was stronger (although not significantly different) in hierarchical state religions than in non-hierarchical state religions or in hierarchical non-state religions. Therefore, it provided no significant support for hypothesis H3c.

To examine hypotheses H4a and hypothesis H4b, we introduced a comprehensive set of control variables, including institutional and normative variables (presented in Table 3C, Table 3Dc and 3d). The significant institutional variables were economic freedom scores, democracy scores, GDP per capita (which we report only in one specification due to potential multicollinearity) and being a former colony of Spain (while we initially included all former colony or former socialist dummies, only the former colony of Spain dummy was statistically significant). The significant normative variables were the level of trust in other people, participation of women in parliament (a proxy for attitudes toward women), share of urban population, the inequality Gini index (which we present only in one specification due to potential multicollinearity), and population growth. As shown in Table 3c, Christian Orthodox and Sunni Muslim majority dummies remained negatively significant in all specifications. Catholics and Shia Muslims remained negatively significant in only one specification. Both Protestant Christians and Orthodox Christians lost significance in the controlled specifications, offering partial support for hypothesis H4a.

Table 3d, shows that after controlling for institutional and normative variables, both the hierarchical state dummy and religiosity level variables, as well as the anti-hierarchical state dummy and religiosity level variables, remained significant, supporting hypothesis H4a. However, as previously described, in some specifications, the negative correlation between hierarchical religions and the religiosity level of hierarchical religions (when characterized as dominant majority religions) with corruption scores was significantly mediated through both normative and institutional groups of variables, providing partial support for hypothesis H4b.

In Table 4, a summary of the effect of different combinations of hierarchical/non-hierarchical and state/non-state religions on corruption levels is presented. In the uncontrolled specifications, hierarchical religion had the strongest negative association with corruption scores, while state religion also had a negative association (although weaker in magnitude). In the combination of non-hierarchical and non-state religions, religiosity level had no association with corruption scores. However, in the controlled specifications, only the hierarchical state religions exhibit a significant negative association with corruption score (although weaker in magnitude than in the uncontrolled specifications). This finding also supports hypothesis H4b, which posits that a portion of the effect by the hierarchical and/or state religions on corruption is mediated by institutional and normative transmitters.

**Table 4:** Religiosity level in different states and associations with corruption scores.

Religiosity Level in …	Uncontrolled		Controlled	
	Statistically Significant	Direction and Strength	Statistically Significant	Direction and Strength
Hierarchical State Religions	Yes	–	Yes	–
Hierarchical Non-State Religions	Yes	–	No	=
Non-Hierarchical State Religions	Yes	–	No	=
Non-Hierarchical Non-State Religions	No	=	No	=
Protestant (Anti-Hierarchical)	Yes	++	No	=
Catholic (Hierarchical Non-State)	Yes	–	No	=

Note: the number of ‘-’ represents the strength of the effect.

Internal differences between hierarchical religions are evident in their effects on corruption. Specifically, Orthodox Christianity, Sunni Islam, and Shia Islam have a direct effect on corruption, whereas Catholicism only has an indirect effect, which is mediated through institutional and normative control variables (Models C2 and C6 in Table 3c, and Model D3 in Table 3d), or through religiosity level (Models C4 and D6 in Table 3C, Table 3Dc and 3d). Conversely, Protestant Christianity has a positive association with corruption score that is fully mediated by the institutional and normative control variables, with no apparent direct effect. A comparison of the direct versus indirect associations found for all seven religions examined is presented in Table 5.

**Table 5:** Direct and indirect associations of religion and corruption scores.

Religion	Direct Association (Model)	Indirect Association (Model)
Sunni Muslim	Yes/Negative	Yes/Negative
Shia Muslim	Yes/Negative	Yes/Negative
Christians Catholic	Yes/Negative	Yes/Negative
Christians Orthodox	No	Yes/Negative
Christians Protestant	No	Yes/Positive
Hindu	No	No
Buddhist	No	No

* Judaism is a majority only in a signal country; thus, we do not include it in the table. The share of Jews in a country variable was statistically insignificant however, it shares in all countries is very low.

Further examination of the Pearson correlations between religious variables and corruption score sheds more light on the association between religion and corruption (Table 2b). For instance, Sunni Muslim countries are associated with increased religiosity and population growth, decreased trust in other people and share of women in parliament (the norms channel), as well as decreased democracy scores and economic freedom scores (the institutional channel). This association may explain the significantly lower GDP per capita observed in these countries. In contrast, Protestant Christian countries are associated with decreased religiosity and population growth, increased trust in other people and share of women in parliament (the norms channel), as well as increased democracy scores and economic freedom scores (the institutional channel). This association may explain the significantly higher GDP per capita observed in these countries. It is worth mentioning that the interaction analysis between the dominant religious variables and the set of institutional and normative variables did not identify any significant associations.

### 4.1 Robustness tests

To test the robustness of our findings, we included additional control variables. First, we included the variable measuring religious diversity based on the total share of religious minorities. However, this variable was statistically insignificant in all specifications, including those using the religion's share in the population variable and in the specifications using the religion majority dummy variables. This was consistent across the baseline specification (Table 3a) and the specifications controlling for institutional and normative variables (Table 3c). Second, we conducted regressions using the WGI corruption control rank instead of the CPI score in each set of regressions (Table 3A, Table 3B, Table 3C, Table 3Da, 3b, 3c, and 3d) in the last two specifications. The results of these models were overall similar to those using the CPI score variable.

## 5 Discussion and concluding remarks

This paper aims to address a puzzle in the existing literature: while it is commonly believed that all religions promote better moral conduct and ethical behavior on a personal level [32,33,69], recent studies have found a positive association between religiosity levels and corruption levels [29]. To solve this puzzle, the study examines both personal and cultural channels that mediate the role of religion in corruption. Furthermore, it investigates two complementary mediators of the cultural channel: institutional and normative transmitters.

The results show that all four hierarchical religions, particularly Sunni Islam and Orthodox Christianity, are associated with lower corruption scores (higher levels of corruption) in their respective countries. This association operates through their dominant majority religious status (the cultural/institutional channel), rather than through the percentage of people in the country belonging to specific religions (the personal channel). Three of the four hierarchical religions had both direct and indirect associations, while Catholicism had a lower corruption score (higher level of corruption) only in the uncontrolled specification and was characterized only by an indirect association through the institutional and normative variables. Anti-hierarchical Protestant Christianity was associated with higher corruption scores (lower levels of corruption), but the full effect was mediated by the institutional and normative variables. The study shows that most of the positive and negative associations of hierarchical and non-hierarchical religions on corruption scores are mediated through the two cultural transmitters: norms and institutions.

The findings are closely linked and are perfectly in line with both the corruption literature and the modern view of economic development literature. Numerous studies have stressed the relationship of institutions and corruption and especially the effective separation of powers, the freedom of the press to monitor the government, and need for transparency and accountability of government to tackle corruption. Other studies stress the role of cultural factors play in corruption, and especially in societies characterized by high of power distance and collectivism (see in more details the literature review section). In parallel, the modern view of economic development, which suggests that institutions give rise to economic growth [70], while culture shapes institutions [71,72]. Our results connect these separate fields in the economic literature by claiming that religion shapes the cultural norms which affects corruption which in turn shapes institutions which finally affect economic development.

Furthermore, despite the inclusion of numerous control variables in the specifications, the impact of three out of four hierarchical religions on corruption scores appears to be directly associated, rather than solely mediated through the effect on institutions and norms. These findings indicate that the analysis of hierarchical versus non-hierarchical religions fails to provide a complete understanding of the issue at hand. The research highlights the significance of a missing parameter, i.e., state religion, where Catholicism and Protestant Christianity, as non-state religions, only exhibit an indirect impact, whereas three hierarchical state religions - Sunni Islam, Shia Islam, and Orthodox Christianity - display both direct and indirect associations with corruption scores. In contrast, two state non-hierarchical religions, Hinduism and Buddhism, do not exhibit any significant associations at all.

The extensive control variables, particularly the economic and institutional variables, and the robustness of the results across alternative specifications, rule out potential alternative explanations or confounding factors. However, the cross-sectional nature of the dataset prevents us from distinguishing between cause and effect. Specifically, we cannot determine whether hierarchical religions in a state shaped cultural norms and institutions that influenced corruption or whether states adopted hierarchical religions that suited their needs during development. Although our results do not directly lead to conclusions, they provide a potential indication. Since many of the countries in our dataset are twentieth-century countries that adopted their religions centuries before, it is possible that religion is the cause and not the outcome. Future research could use more detailed data on corruption perceptions in areas that have switched religions, such as between Catholicism and Protestant Christianity or Sunni Islam and Shia Islam, to shed more light on this important topic. In addition, unfortunately, the data set does not allow exploring the characteristics of state religions that are behind the direct effect such as the level of direct involvement of religious leaders in state affairs, which should be left for future research.

Regarding the personal channel, the study found that the presence of followers of these hierarchical religions in other countries or the presence of followers of non-hierarchical religions, did not correlate with changes in corruption scores. Moreover, recent literature has shown that the negative association between religiosity and corruption scores (positively correlated with levels of corruption) is biased due to omitted variables. Therefore, religiosity is not directly related to corruption scores, but only indirectly related. When religiosity is presented alone, regardless of the dominant religion in the country, it is always significantly negatively associated with corruption score (positively associated with higher levels of corruption). However, when religiosity is multiplied with the dominant majority religious dummies, it is only significantly negatively associated with corruption score for hierarchical and state religions. This suggests that as religiosity levels increase, the religion's members become less critical of the religious leaders and institutions. Thus, in institutional settings that incentivize corruption (for example, hierarchical and state religions), the religiosity level has an indirectly negative impact on corruption score.

This study highlights the liberal view of separating religion from the state for the benefit of both the state and religion. The close interaction between political and religious institutions creates too much concentration of power and decreases incentives to monitor both the government and the religious leaders, ultimately leading to corrupt countries and religious leaders and institutions that do not serve their members.

The practical implications of this study are clear. Checks and balances are crucial in every country, but they are especially vital in countries that have a state hierarchical religion. Furthermore, it is crucial that followers of the state hierarchical religion are knowledgeable about the norms that affect corruption, as well as the transparency and efficacy of state institutions. To combat corruption effectively, a more involved public is needed, especially among followers of the majority religion, who should demand greater transparency in the use of public resources. International agencies that seek to help countries fight corruption should place greater emphasis on promoting transparency in the use of government resources and authority to both the public and the media. They should not rely solely on government self-action to tackle corruption.

## Author contribution statement

Both authors, Prof. Yaron Zelekha and Dr. Gil Avnimelech, equally contributed to all aspects of this paper.

The author contributions are as follows:

Conceived and designed the paper framing and theory: Prof. Yaron Zelekha and Dr. Gil Avnimelech jointly developed the research framing and theory.

Collected the data: Prof. Yaron Zelekha and Dr. Gil Avnimelech both managed the data collection.

Analyzed and interpreted the data: Prof. Yaron Zelekha and Dr. Gil Avnimelech closely collaborated in analyzing and interpreting the data. They performed statistical analyses together, interpreted the results, and drew meaningful conclusions.

Wrote the paper: Prof. Yaron Zelekha and Dr. Gil Avnimelech shared equal responsibility in writing the manuscript. They collaborated on drafting the paper, organizing the content, and refining the language to ensure clarity and accuracy.

Both authors have read and approved the final version of the manuscript and have agreed to be accountable for all aspects of the work, ensuring its integrity and accuracy.

## Data availability statement

Data will be made available on request.

## Funding

This research was supported by grants from the research authority of Ono Academic College. None of these institutions create any conflict of interest.

## Additional information

No additional information is available for this paper.

## Declaration of competing interest

The authors declare that they have no known competing financial interests or personal relationships that could have appeared to influence the work reported in this paper.

## Footnotes

1 https://www.transparency.org/en/what-is-corruption.

2 https://www.worldbank.org/en/news/factsheet/2020/02/19/anticorruption-fact-sheet.
